# From promise to practice: a scoping review of AI applications in abdominal radiology

**DOI:** 10.1007/s00261-025-05144-y

**Published:** 2025-07-28

**Authors:** Anastasia Fotis, Neeraj Lalwani, Pankaj Gupta, Judy Yee

**Affiliations:** 1 Radiology, Albert Einstein College of Medicine, Bronx, USA; 2 Montefiore Medical Center, Bronx, USA; 3 Radiology, Post Graduate Institute of Medical Education and Research, Chandigarh, India

**Keywords:** Abdominal radiology, Artificial intelligence, Classification, Deep learning, Segmentation

## Abstract

AI is rapidly transforming abdominal radiology. This scoping review mapped current applications across segmentation, detection, classification, prediction, and workflow optimization based on 432 studies published between 2019 and 2024. Most studies focused on CT imaging, with fewer involving MRI, ultrasound, or X-ray. Segmentation models (e.g., U-Net) performed well in liver and pancreatic imaging (Dice coefficient 0.65–0.90). Classification models (e.g., ResNet, DenseNet) were commonly used for diagnostic labeling, with reported sensitivities ranging from 52 to 100% and specificities from 40.7 to 99%. A small number of studies employed true object detection models (e.g., YOLOv3, YOLOv7, Mask R-CNN) capable of spatial lesion localization, marking an emerging trend toward localization-based AI. Predictive models demonstrated AUCs between 0.62 and 0.99 but often lacked interpretability and external validation. Workflow optimization studies reported improved efficiency (e.g., reduced report turnaround and scan repetition), though standardized benchmarks were often missing. Major gaps identified include limited real-world validation, underuse of non-CT modalities, and unclear regulatory pathways. Successful clinical integration will require robust validation, practical implementation, and interdisciplinary collaboration.

## Introduction

Artificial intelligence (AI) has facilitated rapid advancements in medicine, as reflected in the over 25-fold increase in FDA clearances (Class II devices) for AI-based medical devices from 2016 to 2023 [1]. This growth is particularly evident in radiology, which has been at the forefront of such expansion, with AI-related PubMed publications on abdominal radiology increasing by a factor of three between 2020 and 2024. Amid these advancements, concerns persist that AI may eventually replace radiologists [2]. However, while this innovation is poised to transform the field, it is unlikely to surpass human expertise entirely, especially in abdominal imaging, where the complexity and variability of diseases challenge the development of algorithms that match the accuracy and adaptability of human readers [3].

Nonetheless, AI’s applications in abdominal radiology are swiftly expanding beyond traditional detection, diagnosis, and classification tasks to include organ segmentation and volumetry, disease staging and progression prediction, and workflow optimization through image reconstruction [1]. Deep learning models, in particular, have demonstrated significant progress in these areas and carry potential implications for daily clinical workflows [4].

AI is also pivotal in the development of imaging biobanks, which enable large-scale data aggregation critical to advancing precision medicine [5]. However, challenges remain, including the low reproducibility of radiomics features in real-world clinical settings [6, 7].

In light of these issues, this scoping review was conducted to systematically map the literature on AI applications in abdominal radiology, which encompassed identifying major research themes; highlighting gaps in validation, clinical integration, and real-world applicability; and providing clear guidance on future research priorities. A scoping review was chosen over systematic evaluations or meta-analyses given the breadth and variability of existing studies. This decision was also justified by the fact that scoping reviews are designed to capture the nature and dimensions of a diverse and rapidly evolving research field without requiring quantitative syntheses or formal quality assessments that are typically associated with systematic reviews or meta-analyses.

## Materials and methods

### Eligibility criteria

The review was directed toward peer-reviewed studies published between 2019 and 2024, written in English, involving human subjects, and assessing AI applications in abdominal radiology. The following works were excluded: preprints, studies published before 2019, non-English publications, nonpeer-reviewed articles, research that evaluated AI performance in diagnostic abdominal radiology, studies focusing on non-abdominal organs (e.g., the thorax, vertebrae, or pelvis) and nonhuman animal models, studies lacking AI performance assessments, and studies whose full texts were inaccessible. No formal evaluation of the quality of the studies or the risk of bias was performed.

### Search strategy and selection of sources of evidence

A comprehensive search was conducted in PubMed in December 2024 using the explicit query “artificial intelligence AND abdominal radiology.” PubMed was selected due to its exhaustive indexing of the biomedical literature and relevance to radiology and AI research.

**Fig. 1:**
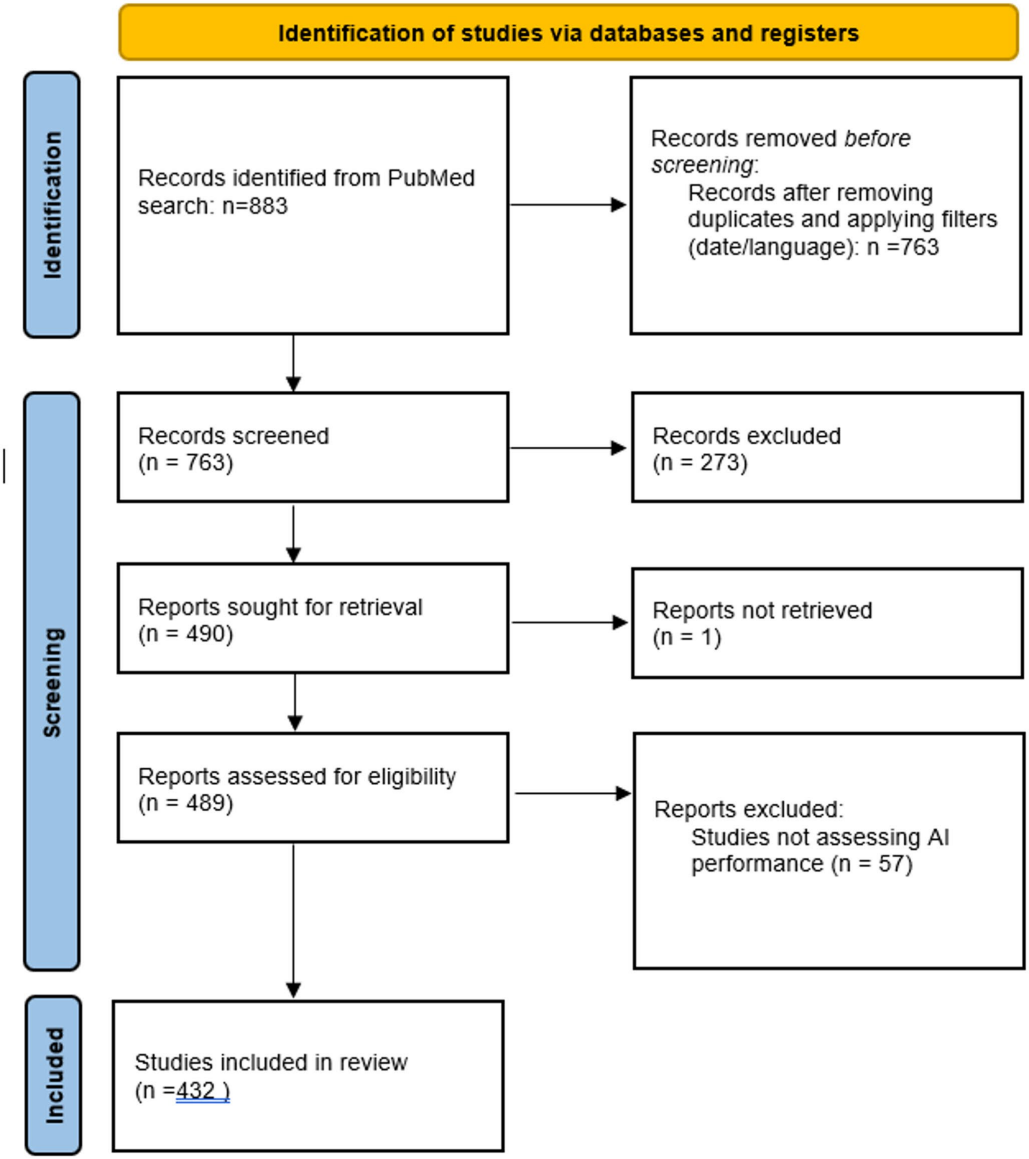
PRISMA-ScR flowchart illustrating the study selection process for the scoping review. The diagram outlines the number of records identified, screened, assessed for eligibility, and ultimately included in the review, following PRISMA-ScR guidelines

Following the PRISMA-ScR guidelines [8], the search initially identified 883 articles, which were reduced to 763 after filtering by language (English) and publication date (the last five years). The abstracts of the 763 studies were screened for eligibility on the basis of the inclusion and exclusion criteria. This round yielded 490 candidate studies, whose full texts were reviewed. Studies that did not explicitly assess AI performance (*n* = 57) and one research whose text was inaccessible were excluded, leaving a final dataset of 432 studies. The selection process was documented using a PRISMA-ScR flowchart (Fig. 1) [8].

### Data charting

Data were systematically charted into a structured spreadsheet that comprised the following categories: authors, publication year, AI model (e.g., CNN, U-Net, random forest), imaging modality (e.g., computed tomography [CT], magnetic resonance imaging [MRI], ultrasound), study characteristics (prospective vs. retrospective), patient demographics, sample size, and applications (segmentation, detection, classification, prediction, workflow optimization). Performance metrics, such as sensitivity, specificity, accuracy, the Dice coefficient, and the area under the curve (AUC), were also recorded. Additional performance metrics (e.g., dose reduction, decreased scan acquisition time, image quality parameters) were documented for workflow optimization studies.

For consistency, studies were categorized under ‘detection’ even if they employed classification models without spatial localization; only five studies utilized true object detection architectures (e.g., YOLOv3, YOLOv7, Mask R-CNN) that generated lesion bounding boxes.

### Data synthesis and presentation

A qualitative descriptive synthesis was conducted to summarize the key research trends, AI applications, validation approaches, and common limitations identified across the studies. The synthesis was aimed at mapping the current research landscape, which included identifying gaps and proposing directions for future research. Consistent with scoping review guidelines, no quantitative meta-analyses or formal statistical analyses, such as funnel plotting or Egger’s regression tests, were performed.

## Results

### Study selection

As previously stated, the search initially identified 883 articles before filtering based on language (English) and publication date reduced the sample to 763. The abstracts of these studies were screened, yielding 490 articles eligible for a full-text review. Inaccessible articles and studies that were not explicitly intended to assess AI performance were excluded, leaving a final dataset of 432 studies. A detailed flowchart documenting this process is presented in Fig. 1.

Characteristics of the sources of evidence

Table 1 summarizes the key characteristics of the reviewed studies on the basis of publication year, type of AI application, and imaging modality.

**Table 1:** Study characteristics and imaging modalities

Study Characteristic	Summary
Publication Year Range	2019–2024
Number of Studies	432 included studies.
Geographic Distribution	Primarily North America, Europe, and East Asia
Imaging Modalities Studied	CT (dominant), MRI (moderate), Ultrasound/X-ray (limited); underrepresentation of ultrasound and X-ray limits modality-wide applicability

Most of the studies focused heavily on segmentation and detection tasks, predominantly employing computed tomography (CT). Research involving MRI was expanding but still limited compared with that revolving around CT. Notably less research involved the use of ultrasound and X-ray modalities.

### AI application mapping

**Fig. 2:**
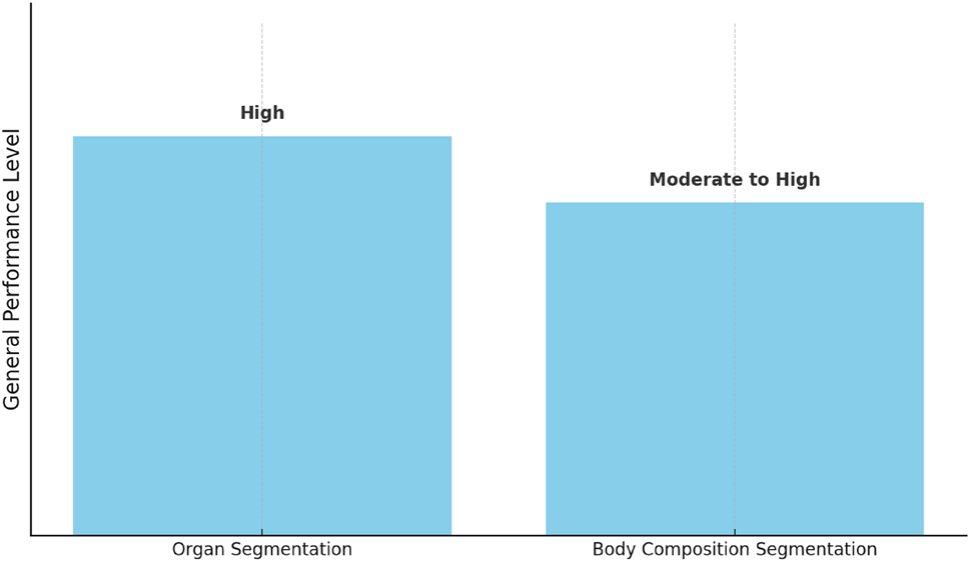
General trends in segmentation AI model performance across two key application areas: organ segmentation and body composition segmentation. Performance levels are summarized qualitatively based on review findings, illustrating high to moderate effectiveness without reference to specific numeric metrics, consistent with scoping review methodology

**Fig. 3:**
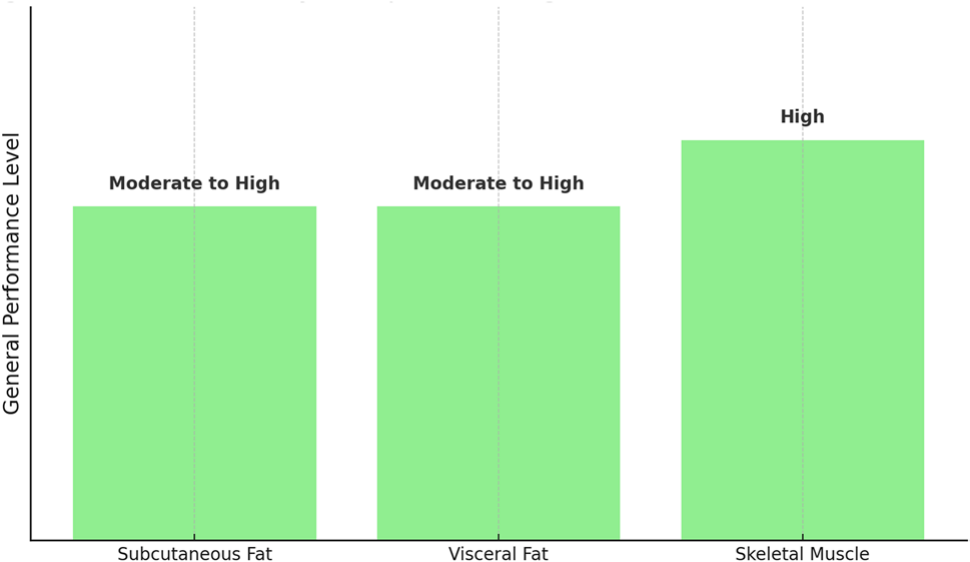
General performance trends of AI models in tumor detection, highlighting qualitative levels for sensitivity, specificity, and area under the curve (AUC). The figure conveys overall effectiveness using broad descriptors (high, moderate to high) without precise numeric values, consistent with scoping review methodology

**Fig. 4:**
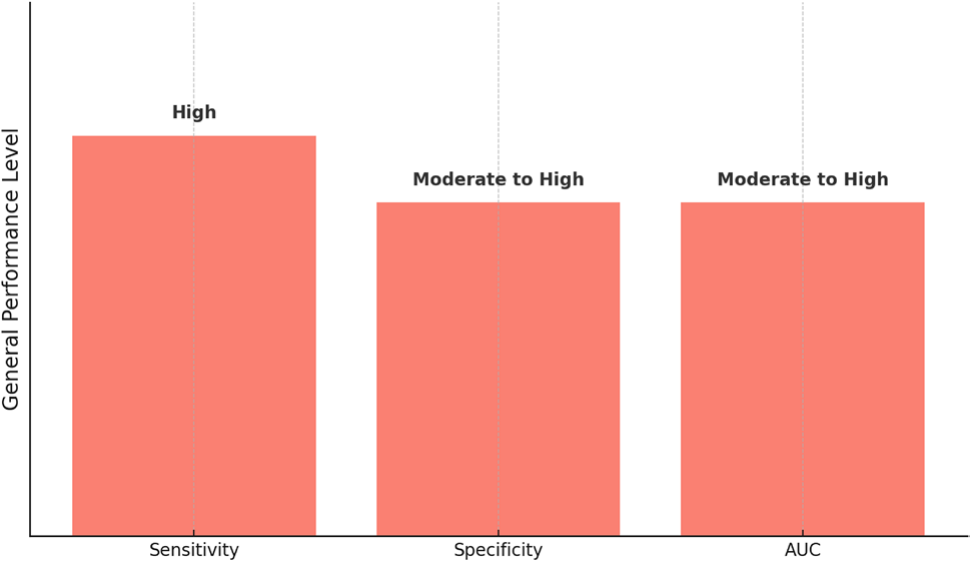
General performance trends of AI models in tumor detection, highlighting qualitative levels for sensitivity, specificity, and area under the curve (AUC). The figure conveys overall effectiveness using broad descriptors (high, moderate to high) without precise numeric values, consistent with scoping review methodology

The examined studies covered four primary areas for AI application: segmentation, detection and classification, prediction, and workflow optimization. Figures 2, 3 and 4 provide visual summaries of performance trends by application area, consistent with scoping review practices emphasizing thematic synthesis. Table 2 summarizes overview of AI Applications in Abdominal Radiology.

**Table 2:** Overview of AI applications in abdominal radiology

AI Application Type	Performance Summary	Common AI Models
Segmentation	High performance in liver and pancreas segmentation; moderate in other organs; reduced accuracy with motion artifacts and low-contrast boundaries.	U-Net, 3D U-Net, CNNs, Transformer hybrids; Dice coefficient range: 0.65–0.90
Classification (with limited use of detection models)	Moderate to high accuracy in tumor detection; variable for inflammatory and rare conditions; ensemble models and multimodal inputs improve performance.	ResNet, DenseNet, VGG, hybrid CNN models; sensitivity 52–100%, specificity 40.7–99%
Prediction	Promising in prognosis and risk stratification; AUCs range from 0.62 to 0.99; strongest when using longitudinal structured data; limited interpretability remains a barrier.	XGBoost, LSTM, deep neural networks; sensitivity 58–100%, specificity up to 95%

*Note: Only 5 of 100 + detection-labeled studies used spatial detection models such as YOLOv3*,* YOLOv7*,* or Mask R-CNN. The majority used classification models without localization capability.*

- Segmentation: AI was extensively used in organ segmentation, particularly in liver [9] and pancreatic imaging [10]. The most frequently reported models were deep learning architectures, such as U-Net [9–11], CycleGAN [12], and hybrid CNN-transformers [13]. While segmentation was widely studied, significant variability existed in dataset size and segmentation accuracy across the evaluated studies.

Segmentation models achieved Dice coefficients of 0.65 [13] to 0.90 [11] and classification models exhibited a sensitivity ranging from 68% [13] to 97% [14] and a specificity of 55% [10] to 100% [15]. For example, a Dice coefficient above 0.85 is typically considered acceptable for liver segmentation in clinical practice. Similarly, classification models achieving sensitivity and specificity above 90% approach the performance thresholds expected for assisting in tumor diagnosis. However, many models fell short of these clinical benchmarks, especially when tested on rare or inflammatory conditions.

deep learning models, such as 3D U-Net, CNN-transformer hybrids, and CycleGAN, performed best in liver [9] and pancreatic segmentation [10]. Nevertheless, accuracy dropped with the presence of low-contrast boundaries, motion artifacts, or overlapping structures.

- Classification and limited detection: The studies on AI-based detection frequently involved tumor identification and lesion detection and localization, for which CNN-based models, such as ResNet-50 [16] and DenseNet [17], were predominantly used. While most studies used classification models that provide only a diagnostic label (e.g., ResNet, DenseNet), five studies employed object detection models capable of lesion localization, including YOLOv3, YOLOv7, and Mask R-CNN.

Classification models, which complete diagnostic characterization tasks, achieved a sensitivity and a specificity ranging from 52% [18] to 100% [17] and 40.7% [19] to 99% [20], respectively, and some studies reported an overall accuracy exceeding 96% [16, 20]. The best performance was observed in models employed to address common pathologies, such as hepatocellular carcinoma [16]. Among these models, the most frequently used were ensemble CNNs, including ResNet-50 and DenseNet, especially in imaging supplemented with clinical data. The detection and classification studies exhibited considerable diversity in terms of pathological complexity and patient populations, but they consistently demonstrated the promising capabilities of AI in addressing prevalent conditions, such as pancreatic tumors [21].

- Prediction: Research on disease progression and outcome prediction covered the growth of aortic aneurysms and the staging of hepatocellular carcinoma, among other processes, with researchers employing models such as XGBoost and hybrid deep learning networks [22, 23]. Disease prediction models exhibited AUCs of 0.62 [24] to 0.99 [25], a sensitivity of 58% [26] to 100% [27], and a specificity of up to 95% [22]. The most commonly used technologies included XGBoost, random forest, and hybrid CNN networks [22, 23, 25]. Predictive accuracy was notably higher in the studies using structured longitudinal datasets than in those employing single time-point inputs. Fewer studies focused on prediction than segmentation or detection and classification, but they highlighted a growing area of AI application in abdominal radiology.
- Workflow Optimization: The studies on workflow optimization primarily explored AI-enhanced image reconstruction, noise reduction, and improved reporting efficiency. These studies (which had sample sizes of 12 to 12,400) [28, 29] reported measurable improvements in image quality and efficiency. AI-assisted systems reduced report turnaround times by 20–30%, decreased repeat scans by 15–25%, and improved urgent case triage by 30–40% [30]. Widely used models included LightGBM and CNN-based reconstruction algorithms [31, 32]. While showing promise, these studies were comparatively fewer than the three discussed above and exhibited a remarkable lack of standardized quantitative metrics. The reporting of quantitative metrics for workplace optimization was also inconsistent.

The gaps identified in the reviewed studies included the limited external validation and real-world testing of AI applications, the insufficient exploration of modalities other than CT imaging, and the lack of comprehensive assessments of AI integration into clinical workflows. The generalizability of the findings was also constrained because the explorations were predominantly single-institution studies, highlighting the need for broader, multicenter research to enhance AI applicability in clinical practice. Table 3 highlights the validation strategies and workflow efficiency impact.

**Table 3:** Validation strategies and workflow efficiency impact

Aspect	Summary
Validation Approach	Predominantly internal validation: fewer than 20% of studies used external or multi-center datasets; limits generalizability and real-world applicability; federated learning is a promising future direction.
Workflow Efficiency Impact	Reported improvements include 20–30% reduction in report turnaround time, 15–25% fewer repeat scans, and 30–40% more efficient triage; however, benefits are inconsistently measured and often lack standardized benchmarks.

## Discussion

AI is poised to revolutionize abdominal radiology, offering advancements in segmentation, detection, classification, prediction, and workflow optimization. Although AI models have shown strong performance across these areas, clinical adoption remains limited due to challenges in validation, generalizability, regulatory approval, and practical implementation [33]. However, instead of reiterating the focus on performance metrics, this discussion centers on the broader implications of AI capabilities and the key barriers that must be overcome to integrate AI into routine clinical practice.

### AI in segmentation: precision versus practical limitations

AI has improved segmentation accuracy in liver, tumor, and pancreatic imaging, significantly reducing the variability associated with manual delineation [9, 10, 16, 21]. Deep learning models, such as U-Net and hybrid CNN-transformers, perform well on high-contrast images, but their performance declines when dealing with low-contrast boundaries, motion artifacts, or overlapping structures [34]. In clinical settings, image quality varies due to patient movement, inconsistent contrast administration, or scanner differences, which can impact AI’s reliability [35]. The overreliance on curated datasets in research raises concerns about how well the results of these models generalize to real-world imaging. Future research should focus on contrast-adaptive AI models and multimodal training approaches that combine CT, MRI, and ultrasound data to improve the robustness of segmentation across different imaging conditions.

### Detection and classification: expanding AI’s capabilities beyond common conditions

Our analysis revealed that the vast majority of studies labeled as ‘detection’ used classification architectures incapable of spatial localization. However, five studies did employ true object detection models—specifically YOLOv3, YOLOv7, and Mask R-CNN—highlighting an emerging but underutilized application of detection-specific algorithms in abdominal imaging. AI-based detection and classification systems, particularly CNN-based models (e.g., ResNet-50 and DenseNet), have shown high sensitivity in detecting tumors and vascular abnormalities, but performance varies significantly depending on the rarity and complexity of pathology [36–40]. AI is constrained when addressing subtle abnormalities, inflammatory conditions, and diseases with high interpatient variability, such as Crohn’s disease, for which imaging features are less distinct [19, 41, 42]. Additionally, radiologists rely not only on image patterns but also on clinical history and laboratory data—factors that have yet to be integrated effectively into AI technologies [43]. Future developments should focus on multisource AI models that encompass imaging data, laboratory results, and patient histories to improve diagnostic accuracy, particularly for complex and rare conditions.

### Prediction models: the challenge of clinical interpretability

The predictive capabilities of AI models, particularly XGBoost and deep learning models, show promise in forecasting disease progression, such as the growth of aortic aneurysms and the stage of hepatocellular carcinoma staging [44–47]. However, predictive accuracy varies depending on data structure, imaging modality, and disease complexity. While AI often outperforms traditional statistical models in dealing with structured datasets, the process underlying decision-making in such technologies remains opaque. Physicians are unlikely to trust AI predictions without clear explanations of how risk scores or prognostic outcomes are determined [48, 49]. Addressing this problem requires a shift to explainable AI models, which provide transparent reasoning behind predictions. Regulatory bodies may also require AI developers to implement explanatory outputs, ensuring that AI recommendations align with clinical reasoning rather than operating as black-box systems.

### Workflow optimization: measurable gains but limited standardization

AI’s impact on workflow optimization is promising but underquantified. The reviewed studies showed 20–30% faster report turnaround times, 15–25% fewer repeat scans, and 30–40% improved triaging efficiency with AI-assisted reporting [30]. The problem is that there are no clear benchmarks for assessing AI’s effectiveness in optimizing workflows, unlike segmentation, detection, and classification, whose performance can be measured using Dice coefficients or sensitivity/specificity levels. Future studies should establish quantitative metrics for workflow efficiency, such as AI’s impact on diagnostic speed, error reduction, and resource allocation. AI must also be seamlessly integrated with existing PACS and RIS systems to ensure that workflow improvements translate into actual clinical benefits. Apart from technical integration with the aforementioned systems, limited regulatory approvals for dynamically evolving AI tools and clinician concerns about black-box model transparency are key implementation challenges [48–50]. Additionally, the economic feasibility of deploying AI—particularly in small institutions—is complicated by the high initial costs incurred from training, infrastructure upgrades, and ongoing maintenance, among others [51]. Without sustainable business models or external funding, widespread implementation may remain out of reach despite promising results.

### Validation and generalizability: the weakest links in AI adoption

A major barrier to AI implementation is the reliance on internal validation rather than multicenter verification studies. Of the more than 220 validation studies reviewed, fewer than 20% externally validated AI outputs, raising concerns about real-world applicability and generalizability across diverse clinical environments. Multicenter validation and federated learning approaches are critical next steps in mitigating overfitting and enhancing trust in AI systems. Models trained on single-institution datasets often perform well in controlled research settings but fail to generalize across different scanners, patient demographics, and imaging protocols [52, 53]. A promising approach to improving AI robustness in terms of generalizability and reduced overfitting is federated learning, in which AI models are trained across multiple institutions and real-world clinical variability without sharing raw patient data [54, 55]. An important consideration, however, is that federated learning requires collaborative infrastructure, which is often hindered by data privacy regulations and proprietary restrictions [55].

### Regulatory and ethical barriers: ai’s path to approval

The absence of FDA clearance (primarily for Class II devices) or equivalent regulatory approvals remains a primary deterrent to AI deployment in clinical settings [56, 57]. As previously stated, the over 220 studies assessed mostly relied on internal validation, risking overfitting, and multicenter trials were underutilized. Unlike traditional imaging software, AI operates dynamically, pointing to the need for regulatory agencies to establish new evaluation frameworks that account for the technology’s evolving nature. One approach is building a tiered approval system, in which AI models are first deployed in controlled environments before full clinical integration. Additionally, ethical concerns surrounding AI accountability in misdiagnoses must be addressed. Legislation is unclear as to determining liability when an AI system issues erroneous diagnoses—whether this falls on the AI developer, the hospital, or the interpreting radiologist [58, 59]. Implementing human-in-the-loop AI frameworks, wherein AI assists rather than replaces radiologists, may offer a practical regulatory pathway that balances AI’s potential with the need for physician oversight.

### The future and feasibility of AI in abdominal radiology

Although AI research continues to expand, much of it remains focused on CT imaging, especially for liver and renal applications, with limited adoption in MRI, ultrasound, and X-ray imaging. This gap may be attributable to technical limitations, such as operator dependency in ultrasound and lower soft-tissue contrast in X-ray, which challenge standardized data acquisition and algorithm training. These limitations reduce reproducibility and hinder large-scale dataset creation, potentially explaining the relative scarcity of AI applications in these modalities. The orientation towards CT reflects the availability of large, standardized CT datasets but restricts the development of AI tools that are applicable across more variable or operator-dependent modalities, such as ultrasound. This imbalance also suggests that AI development prioritizes modalities with clear anatomical structures. This deficiency can be addressed through multimodal AI, which integrates data from multiple imaging modalities, thus improving diagnostic accuracy across different technologies. Another concern is economic feasibility. Although AI reduces reporting times, minimizes repeat scans, and enhances triaging efficiency [30], the high costs of PACS integration, AI model training, implementation, maintenance, and compliance with HIPAA/FDA clearance regulations add upfront financial burdens and potentially inhibit adoption, particularly in small healthcare institutions [53]. Offsetting these costs may require institutions to acquire subscription-based, pay-per-use AI services or government grants. Future studies should explore the long-term cost-effectiveness of AI-driven radiology, assessing whether AI investment leads to sustained improvements in diagnostic efficiency and patient outcomes.

### Limitations

While this study provides valuable insights into the current landscape of AI in abdominal radiology, several limitations must be acknowledged. As a scoping review, no formal quality or risk-of-bias assessment was conducted, and coverage was restricted to peer-reviewed, English-language publications indexed in a single database (PubMed). PubMed was selected due to its comprehensive coverage of biomedical and radiology-specific literature. However, this may have limited the retrieval of studies indexed exclusively in other databases such as EMBASE or Scopus. Nonetheless, given the clinical and radiology-focused scope of this review, most relevant studies were likely captured through PubMed, which indexes leading journals in abdominal imaging and medical AI. This approach aligns with accepted scoping review methodology, where transparency and topic relevance guide database selection. These decisions may still have affected the comprehensiveness and generalizability of the findings. Additionally, a few potentially relevant studies were inaccessible due to technical issues. A major challenge also lay in the inconsistency of AI model evaluations across the examined studies.

Without standardized metrics, such as accuracy, sensitivity, specificity, AUCs, or Dice coefficients, direct comparisons between models were difficult, hindering definitive conclusions about performance. This issue was compounded by the validation practices carried out in the evaluated studies, of which the majority relied heavily on internal verification, thereby increasing the risk of overfitting and reducing real-world applicability. Fewer than 20% conducted external or multicenter validation—an essential step for assessing AI generalizability across diverse populations and clinical settings. Most AI models were also trained on retrospective data, with minimal longitudinal validation conducted. This shortcoming prevented a definitive clarification of how well these tools can track disease progression over time.

A notable concern as well is modality bias. The studies centered primarily on CT liver and renal imaging, while MRI, ultrasound, and X-ray were underexplored. This imbalance reflects the availability of standardized CT datasets but restricts the broader applicability of AI across more variable imaging modalities. Moreover, training data often lacked demographic transparency, raising ethical concerns about potential performance disparities in underrepresented patient groups.

Furthermore, claims about workflow efficiency gains from AI were largely speculative. While many studies demonstrated reduced turnaround times, decreased repeat scans, and improved triage [30], few conducted a standardized or quantitative validation of these benefits. While a few studies applied object detection models (*n* = 5), most ‘detection’ studies used classification models without spatial localization, limiting the scope of lesion detection evaluation in this review. Lastly, the economic feasibility of AI implementation—including infrastructure costs, scalability, and sustainability—was beyond the scope of this review and remains underexplored in the current literature.

Together, these limitations highlight the need for more rigorous, multi-institutional, and methodologically transparent studies to fully evaluate AI’s clinical utility, equity, and sustainability in abdominal radiology.

## Conclusion

This scoping review mapped existing scholarship on AI applications in abdominal radiology, summarizing broad research themes, identifying key research gaps, and suggesting directions for future investigation. AI is revolutionizing abdominal radiology by enhancing classification, segmentation, prediction, and workflow efficiency, with early but limited integration of true detection (localization) models in a few studies.

Rather than providing detailed performance outcomes, this review highlighted the current landscape of AI applications and the limitations that must be overcome for real-world integration. AI should serve as a decision support tool rather than replace radiologists—a goal that necessitates regulatory approvals, multicenter validation, and standardized performance benchmarks for clinical reliability. Beyond ensuring accuracy, the technology’s impact on workflow optimization must be quantified, while financial sustainability must be evaluated using viable cost models.

Looking forward, AI-driven personalized medicine promises precision diagnostics, but overcoming the barriers identified in this work is essential for translating AI’s potential into real-world clinical practice.

## Data Availability

No datasets were generated or analysed during the current study.
